# SLEEP QUALITY, COGNITIVE AGING, AND MORTALITY AMONG MIDDLE TO OLDER AGED US ADULTS

**DOI:** 10.1093/geroni/igae098.3720

**Published:** 2024-12-31

**Authors:** Rio Tate, May Beydoun

**Affiliations:** University of South Florida, Tampa, Florida, United States; National Institute on Aging, Baltimore, Maryland, United States

## Abstract

**Background:**

Sleep and dementia may interact with each other to determine mortality risk, perhaps differentially by sex and by race.

**Methods:**

The study examined bi-directional associations of dementia, sleep and mortality, while testing both interactions and mediating effects in a large sample of older US adults using the nationally representative Health and Retirement Study (HRS) dataset, and middle-aged US adults using the Healthy Aging in Neighborhoods of Diversity across the Life Span (HANDLS) dataset. 6,911 older participants and 1,364 middle-aged participants were included for analysis.

**Results:**

Regarding HRS, poor sleep quality was associated with higher mortality risk with a dose response relationship, and a strong association between dementia status and mortality risk was found. Dementia status’ positive association with mortality risk was stronger in the “better sleep” group (lowest tertile of “poor sleep” variable), with significant interaction between dementia status and poor sleep tertile. Regarding HANDLS, poorer sleep was associated with lower survival probability over time compared to better sleep, particularly among individuals with above median global mental status.

**Conclusions:**

Poor cognition was positively associated with mortality risk, particularly among males and among White older adults, and individuals with better sleep quality in both HANDLS and HRS studies. Future studies should uncover some of the underlying mechanisms behind the dementia-sleep antagonistic interactions.

